# Supplementary material for: Reconstructing the sediment concentration of a giant submarine gravity flow
Source: Nat Commun. 2018 Jul 5;9:2616. doi: 10.1038/s41467-018-05042-6 (PMC6033887; doi:10.1038/s41467-018-05042-6)
Supplement: Supplementary file 1 — Supplementary Information [file 41467_2018_5042_MOESM1_ESM.pdf]

Supplementary information

Reconstructing the sediment concentration of a giant submarine gravity flow

Stevenson et al.

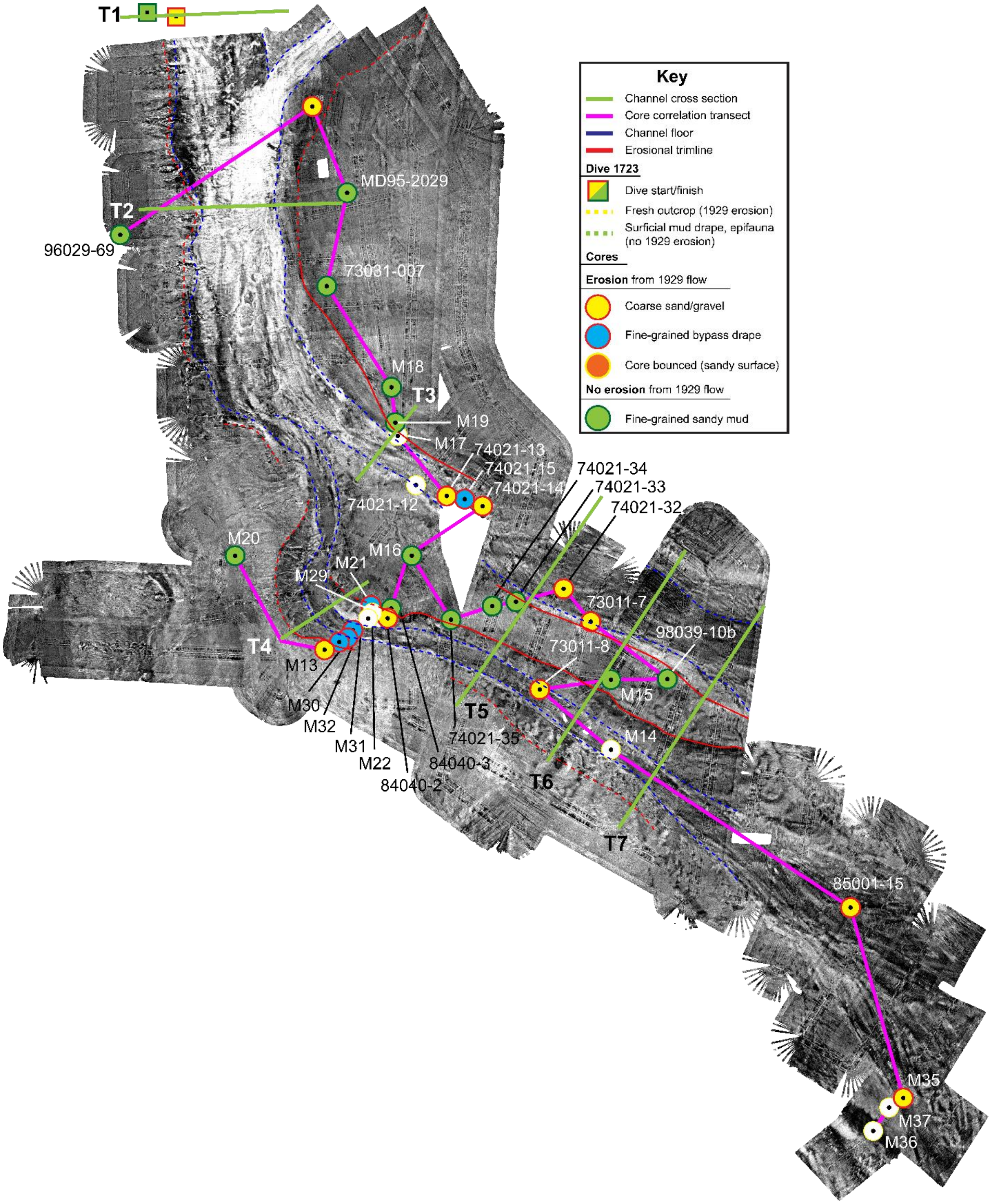

Supplementary Figure 1. Seafloor acoustic backscatter map across the lower Eastern Valley channel system, off shore Grand Banks. Bathymetry data was collected aboard

RV Maria S. Merian Cruise MSM47 between 30/09/2015 and 30/10/2015. Cores are a mix of recently recovered sediment cores aboard Cruise MSM47 (M-cores) and archive material from the Bedford Institute of Oceanography. All cores are labelled and are coloured to represent the different stratigraphic record of the 1929 flow (see key): the erosive parts of the flow are characterized by either thick (metres) deposits of coarse-grained sands and gravels, or sharp erosional surfaces draped by mud, and; the non-erosive parts of the flow are recorded by a thin (centimetres) fine-grained sandy mud. Core correlation transect is highlighted in pink with details shown in supplementary Figures 2A and 2B.

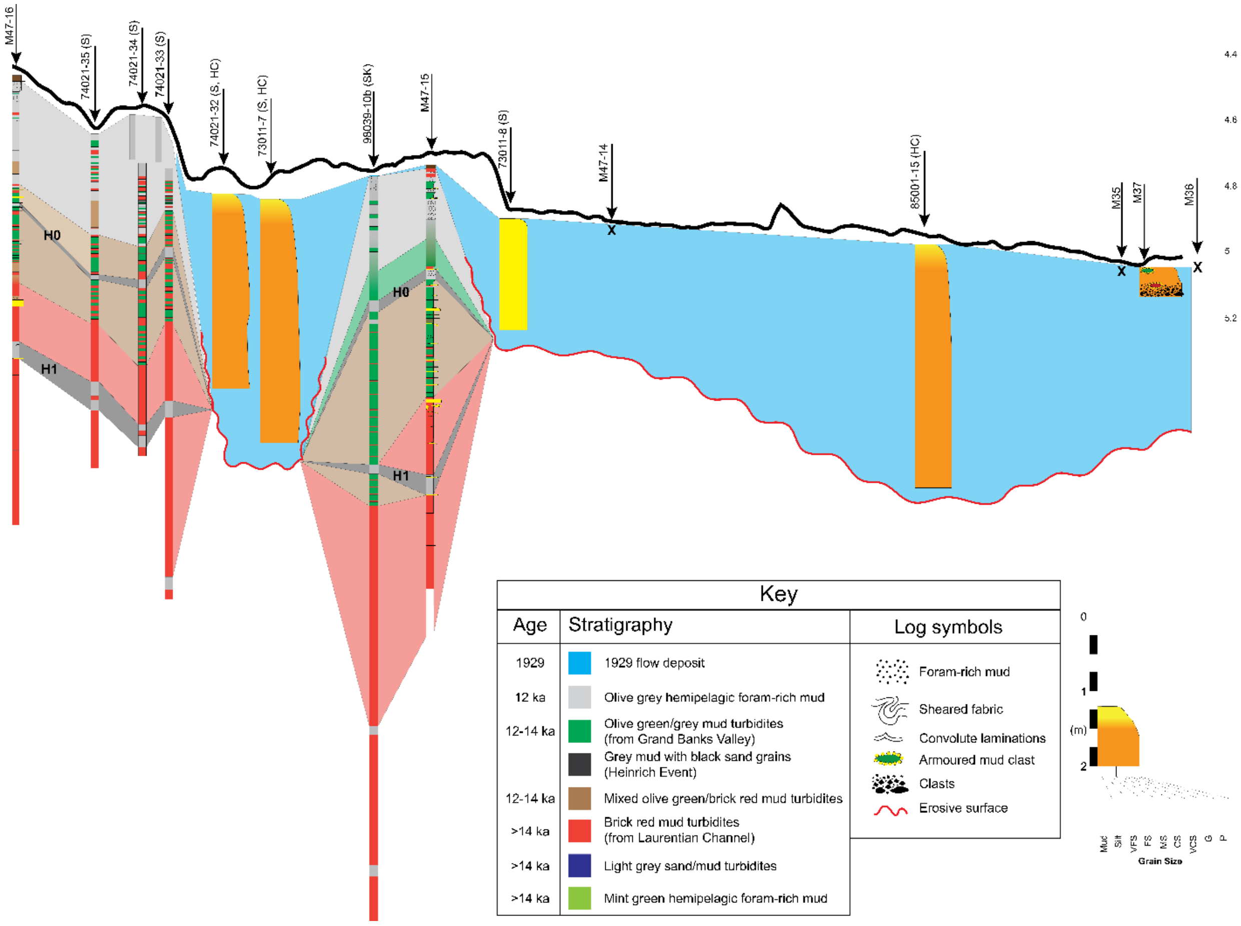

(A)

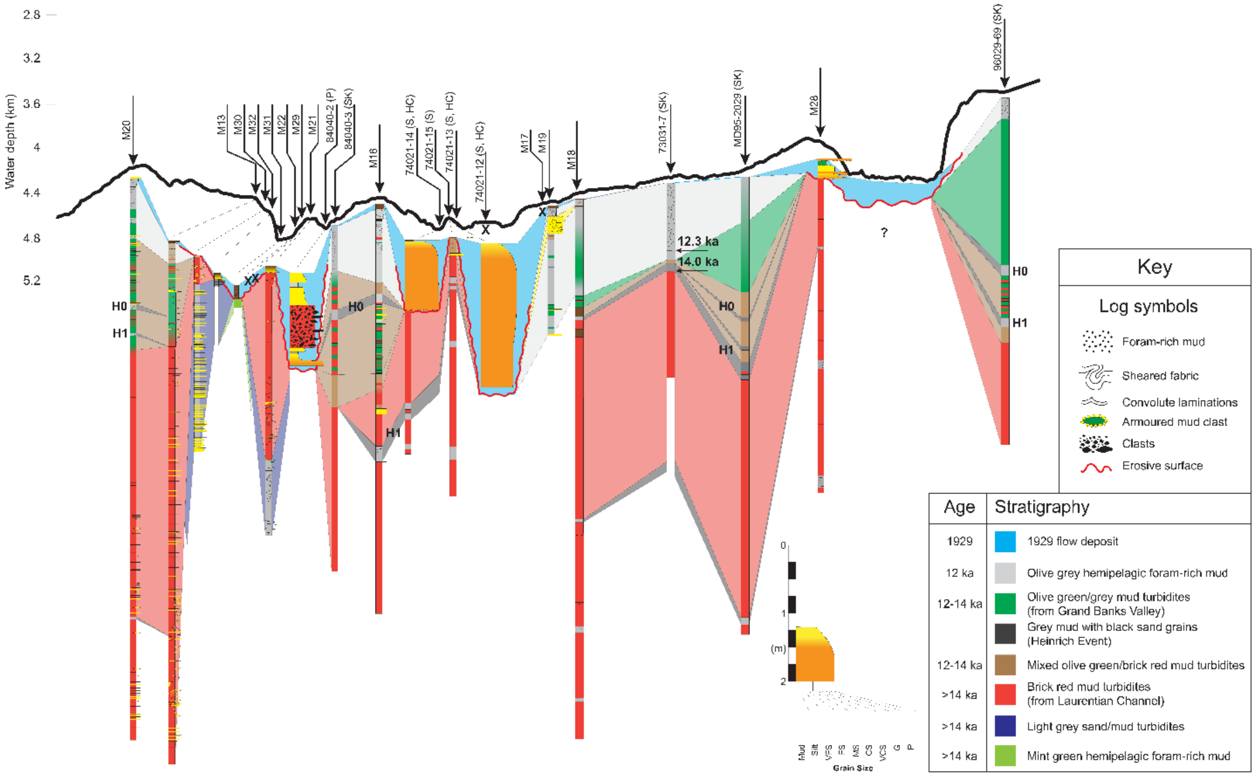

(B)

Supplementary Figure 2. Core correlation of stratigraphy across the Grand Banks slope. Part (A) shows the upper slope and part (B) the lower slope stratigraphy. Cores are a mix of recently recovered sediment cores aboard the Maria Merian Cruise MSM47 (M-cores) and archive material from the Bedford Institute of Oceanography. Archive material is adapted from various sources: (S)[1], (HC)[2], (SK)[3], and (P)[4]. Stratigraphic correlations and ages of sediments are based on SK. The uppermost part of the stratigraphy is an olive-grey foram-rich mud, which forms a regional hemipelagic drape across the Grand Banks

slope. The erosional extent of the 1929 flow is determined by the presence of this hemipelagic drape across the slope: if missing then significant erosion has occurred, or if present then no erosion has occurred. The depth of erosion is difficult to constrain, however, it appears to range from several metres across the channel margins to several 10's of metres in parts of the channel floor.

Supplementary References

1. Stow, D. A. V. Laurentian Fan: Morphology, sediments, processes, and growth pattern. *Am. Assoc. Pet. Geol. Bull.* **65,** 375–393 (1981).
2. Hughes-Clarke, J. E. The geological record of the 1929 Grand Banks earthquake and its relevance to deep-sea clastic sedimentation. *PhD Thesis, Dalhousie Univ. Halifax, Nov. Scotia.* (1988).
3. Skene, K. I. & Piper, D. J. W. Late Quaternary stratigraphy of Laurentian Fan: A record of events off the eastern Canadian continental margin during the last deglacial period. *Quat. Int.* **99–100,** 135–152 (2003).
4. Piper, D. J. W., Shaw, J. & Skene, K. I. Stratigraphic and sedimentological evidence for late Wisconsinan sub-glacial outburst floods to Laurentian Fan. *Palaeogeogr. Palaeoclimatol. Palaeoecol.* **246,** 101–119 (2007).
